# Regulation of T cell alloimmunity by PI3Kγ and PI3Kδ

**DOI:** 10.1038/s41467-017-00982-x

**Published:** 2017-10-16

**Authors:** Mayuko Uehara, Martina M. McGrath, Shunsuke Ohori, Zhabiz Solhjou, Naima Banouni, Sujit Routray, Catherine Evans, Jonathan P. DiNitto, Abdallah Elkhal, Laurence A. Turka, Terry B. Strom, Stefan G. Tullius, David G. Winkler, Jamil Azzi, Reza Abdi

**Affiliations:** 1 Transplantation Research Center, Renal Division, Brigham and Women’s Hospital and Harvard Medical School, 221 Longwood Avenue, Boston, MA 02115 USA; 2 Infinity Pharmaceuticals, Inc 784 Memorial Drive, Cambridge, MA 02139 USA; 3 Division of Transplant Surgery and Transplant Surgery Research Laboratory, Brigham and Women’s Hospital and Harvard Medical School, 75 Francis Street, Boston, MA 02115 USA; 4 Center for Transplantation Sciences, Massachusetts General Hospital/Harvard Medical School, Massachusetts Massachusetts General Hospital-East Charlestown Navy Yard Building 149, 13th Street, Charlestown, MA 02129-2020 USA; 5 The Transplant Institute, Beth Israel Deaconess Medical Center/Harvard Medical School, 330 Brookline Avenue, E/CLS Room 607, Boston, MA 02215 USA

## Abstract

Phosphatidylinositol-3-kinases (PI3K) γ and δ are preferentially enriched in leukocytes, and defects in these signaling pathways have been shown to impair T cell activation. The effects of PI3Kγ and PI3Kδ on alloimmunity remain underexplored. Here, we show that both *PI3Kγ* ^*−/−*^ and *PI3Kδ* ^*D910A/D910A*^ mice receiving heart allografts have suppression of alloreactive T effector cells and delayed acute rejection. However, *PI3Kδ* mutation also dampens regulatory T cells (Treg). After treatment with low dose CTLA4-Ig, *PI3Kγ* ^*−/−*^, but not *PI3Κδ* ^*D910A/D910A*^, recipients exhibit indefinite prolongation of heart allograft survival. *PI3Kδ* ^*D910A/D910A*^ Tregs have increased apoptosis and impaired survival. Selective inhibition of PI3Kγ and PI3Kδ (using PI3Kδ and dual PI3Kγδ chemical inhibitors) shows that PI3Kγ inhibition compensates for the negative effect of PI3Kδ inhibition on long-term allograft survival. These data serve as a basis for future PI3K-based immune therapies for transplantation.

## Abstract (web summary)

Phosphatidylinositol-3-kinases (PI3K) γ and δ are key regulators of T cell signaling. Here the author show, using mouse heart allograft transplantation models, that PI3Kγ or PI3Kδ deficiency prolongs graft survival, but selective inhibition of PI3Kγ or PI3Kδ reveals alternative transplant survival outcomes post CTLA4-Ig treatment.

## Introduction

Current immunosuppressive drugs (ISD) commonly suppress both effector and regulatory axes of adaptive immunity and fail to induce immune regulation, which is critical for long-term graft acceptance. Furthermore, ISDs contribute to microvascular toxicity and organ failure, as well as major long-term complications such as malignancy, metabolic disorders, and infections^1, 2^. Therefore, the development of targeted immunomodulatory agents with improved efficacy and safety profiles^3^ in solid organ transplantation is highly desirable.

PI3Ks belong to the family of lipid kinases that phosphorylate the 3′OH-group of phosphatidylinositols to generate phosphatidylinositol-3,4,5-triphosphate (PtdIns(3,4,5)p3), which then interacts with the pleckstrin-homology (PH)-domains of various signal transduction proteins including AKT^4, 5^. Class I PI3Ks are the best characterized of the different PI3K classes and are composed of regulatory subunits (p85) and catalytic subunits (p110α, p110β, p110δ, and p110γ). Class I PI3Ks are further classified as class IA or IB according to their modes of activation: class IA PI3Ks are activated downstream of tyrosine kinase receptors, whereas the class IB PI3K has only one subunit (PI3Kγ) and is activated by G protein-coupled receptors^5, 6^.

The γ and δ catalytic forms of PI3K are preferentially enriched in leukocytes and, through their capacity to regulate the function of immune cells^5, 7–10^, represent a promising drug target for the treatment of inflammatory diseases. Although PI3Kγ is activated by G protein-coupled receptors including the chemokine receptors, our data and others indicate that PI3Kγ-deficient T cells also have a diminished anti-CD3 proliferative response^11–15^. The importance of p110γ in alloimmune responses also lies in its capacity to regulate innate immune cells and inflammatory responses^8^. PI3Kδ is a sister isoform and lies downstream of tyrosine kinase-associated receptors, T cell receptor (TCR), co-stimulatory and cytokine receptors^16–22^. The lack of PI3Kδ is detrimental to effector T cells (Teff)^23, 24^.

Despite an accumulating body of data on the role of PI3Kγ and δ in immunity, the mechanisms by which the PI3Kγ and δ signaling pathways control alloimmune responses remains to be explored. Here, we show for the first time the role of PI3Kγ and PI3Kδ pathways in determining the fate of alloimmune responses.

## Results

### PI3Kγ or PI3Kδ deletion suppresses T cell alloreactivity

**Fig. 1:**
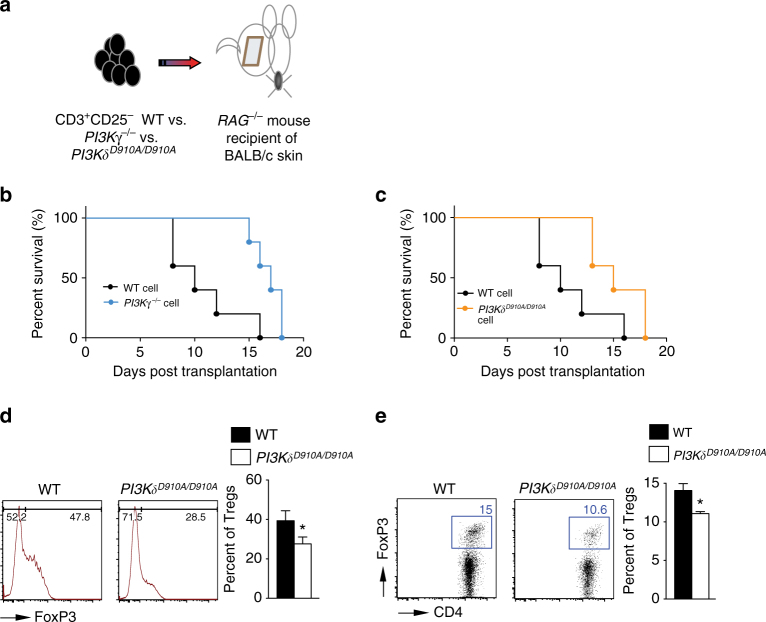
PI3Kγ or PI3Kδ deletion impairs T cell activation in vivo. **a** Schematics describing the methodology used in this experiment. *RAG* ^*−/−*^ mice received a BALB/c skin allograft; on day 1 post-transplant, the mice were injected with 6 × 10^6^ CD3^+^CD25^−^ T cells isolated from splenocytes of *PI3Kγ* ^*−/−*^, *PI3Kδ* ^*D910A/D910A*^ or WT naive mice. **b** Graft survival in recipients of *PI3Kγ* ^*−/−*^ T cells significantly exceeded that in recipients of control T cells (MST of 17 vs. 10 days, respectively, *p < 0.05, *t*-test, *n* = 5/group). **c** Graft survival in recipients of *PI3Kδ* ^*D910A/D910A*^ T cells significantly exceeded that in recipients of control T cells (MST of 15 vs. 10 days, respectively, *p < 0.05, *t*-test, *n* = 5/group). **d** Representative figures of flow cytometry analysis of splenocytes retrieved from transplanted mice at day 7 post-transplant. Data shows significant decrease in Treg induction in the DLN of *PI3Kδ* ^*D910A/D910A*^ recipients of BALB/c skin and WT controls (*p < 0.05, *t*-test, *n* = 3/group). Bar graph represents the percentage of Tregs. **e** Representative example of FACS staining of CD4^+^ *PI3Kδ* ^*D910A/D910A*^ and WT T cells stimulated with anti-CD3/CD28 Abs in the presence of IL-2 and TGFβ. (Data are representative of three separate experiments, *p < 0.05, *t*-test, *n* = 3–4/group). Bar graph represents the percentage of induced Tregs. **d**, **e** The *graphs* show data as mean ± s.e.m. (MST mean survival time; Treg regulatory T cell)

To study the effect of PI3Kγ and PI3Kδ deletion on alloimmune responses in vivo, we injected 6 × 10^6^ CD3^+^CD25^−^ T cells isolated from splenocytes of *PI3Kδ* ^*D910A/D910A*^, *PI3Kγ* ^*−/−*^, or WT naive mice, into *RAG* ^*−/−*^ recipients of BALB/c skin allograft at day 1 post-transplant (Fig. 1a). Graft survival in recipients of *PI3Kγ* ^*−/−*^ or *PI3Kδ* ^*D910A/D910A*^ T cells significantly exceeded that in recipients of control T cells (*p < 0.05, *t*-test, *n* = 5/group) (Fig. 1b, c). Interestingly, there was a significant decrease in regulatory T cell (Treg) induction in mice receiving *PI3Kδ* ^*D910A/D910A*^ compared to WT CD3^+^CD25^−^ T cells (27.60% ± 4.07% vs. 39.35% ± 5.05%, respectively, *p* = 0.05, *t*-test, *n* = 3/group) (Fig. 1d). Similarly, under Treg polarizing conditions, there was a marked decrease in Treg induction in vitro in the *PI3Kδ* ^*D910A/D910A*^ group compared with WT (11.07% ± 0.26% vs. 14.07% ± 0.88%, respectively, *p < 0.05, *t*-test, *n* = 3–4/group) (Fig. 1e).

### PI3Kγ or PI3Kδ deletion impairs T cell activation in vitro

*PI3Kγ* ^*−/−*^ splenocytes proliferated significantly less when stimulated with allogeneic cells, as measured by thymidine incorporation (Supplementary Fig. 1A). Similarly, alloantigen stimulation of *PI3Kγ* ^*−/−*^-deficient T cells led to a significantly lower frequency of interferon (IFN)γ producing T cells than WT controls (Supplementary Fig. 1B). *PI3Kδ* ^*D910A/D910A*^ CD4^+^ and CD8^+^ T cells also proliferated significantly less than WT T cells upon stimulation with anti-CD3 and anti-CD28 antibodies, as measured by thymidine incorporation (Supplementary Fig. 1C, D).

### PI3Kγ or PI3Kδ deletion prolongs heart allograft survival

**Fig. 2:**
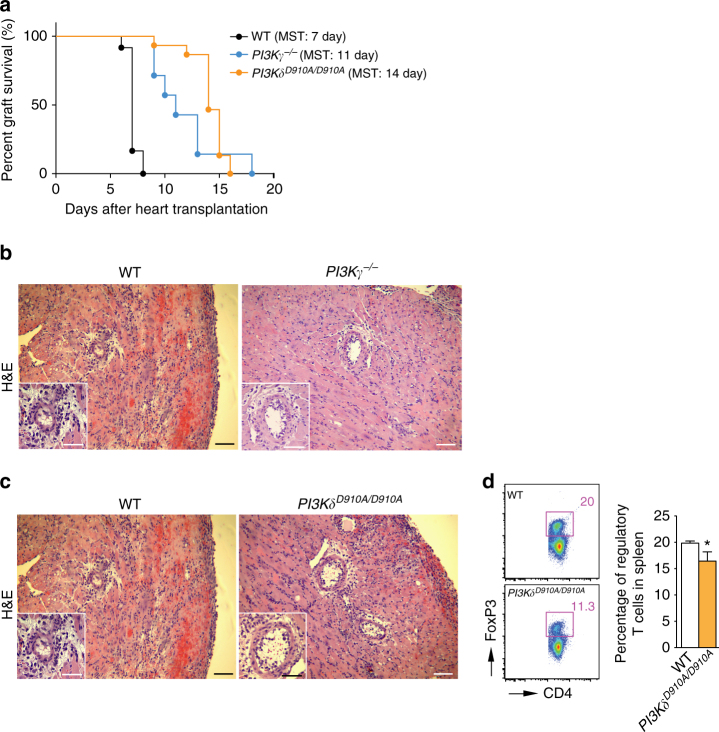
PI3Kγ or PI3Kδ inhibition suppresses acute rejection. Naive BALB/c heart allografts were transplanted into fully allogeneic C57BL/6 (WT), *PI3Kγ* ^*−/−*^, or *PI3Kδ* ^*D910A/D910A*^ recipients. **a** *PI3Kγ* ^*−/−*^ recipients exhibited prolonged allograft survival compared to WT recipients (MST of 11 vs. 7 days respectively, *p < 0.05, *t*-test, *n* = 10/group). *PI3Kδ* ^*D910A/D910A*^ recipients of BALB/c hearts showed significant prolongation of heart allograft survival compared to control (MST of 14 vs. 7 days, respectively, *p < 0.05, *t*-test, *n* = 10–14/group). **b** Representative examples of H&E staining of cardiac allograft histology show well-preserved myocytes and lower cellular inflammatory infiltrate at 7 days post-transplant in the *PI3Kγ* ^*–/–*^ mice compared to WT controls (*Scale bar* 50 μm, inset *scale bar* 25 μm). **c** Representative examples of cardiac allograft histology show well preserved myocytes and lower cellular inflammatory infiltrate at 7 days post-transplant in the *PI3Kδ* ^*D910A/D910A*^ mice compared to WT controls (H&E stain, *scale bar* 50 μm, inset *scale bar* 25 μm). **d** Representative dot plots of Tregs (CD4^+^FoxP3^+^) in splenocytes of WT and *PI3Kδ* ^*D910A/D910A*^ recipients at day 7 post-transplant. Bar graph represents the percentage of Tregs in splenocytes in *PI3Kγ* ^*−/−*^ recipients compared to WT at day 7 post-transplant (*p < 0.05, *t*-test, *n* = 6/group, the graph shows data as mean ± s.e.m.). (MST mean survival time, Treg regulatory T cell)

We first used a model of acute cardiac transplant rejection as a preclinical model to study the immunosuppressive role of PI3Kγ and PI3Kδ deletion. Naive BALB/c heart allografts were transplanted into fully allogeneic C57BL/6 (WT) recipients, *PI3Kγ* ^*−*/*−*^ C57BL/6 (*PI3Kγ* ^*−*/*−*^) recipients or *PI3Kδ* ^*D910A/D910A*^ C57BL/6 (*PI3Kδ* ^*D910A/D910A*^) recipients. *PI3Kγ* ^*−*/*−*^ and *PI3Kδ* ^*D910A/D910A*^ recipients exhibited prolonged allograft survival compared to WT recipients (mean survival time, (MST) *PI3Kγ* ^*−*/*−*^ vs. WT: MST of 11 vs. 7 days, respectively, *p < 0.05, *t*-test, *n* = 10/group, *PI3Kδ* ^*D910A/D910A*^ vs. WT: MST of 14 vs. 7 days, respectively, *p < 0.05, *t-*test, *n* = 10–14/group) (Fig. 2a). Accordingly, *PI3Kγ* ^*−*/*−*^ and *PI3Kδ* ^*D910A/D910A*^ recipients showed marked reduction in the severity of acute rejection of the heart allograft as assessed by histological analysis (Fig. 2b, c). We also observed a significant decrease in the number of CD4^+^ and CD8^+^ effector cells (CD44^High^CD62L^Low^) in the secondary lymphoid tissues of *PI3Kγ* ^*−/−*^ recipients compared to WT (at day 7 post-transplant) (*p < 0.05, *t*-test, *n* = 6/group) (Supplementary Fig. 2A–D). Furthermore, *PI3Kγ* ^*−/−*^ recipients showed reduced T cell proliferative responses and suppression of inflammatory cytokine production at 7 days post-transplantation as assessed by MLR and luminex-assays (*p < 0.05, *t*-test, *n* = 4–6/group) (Supplementary Fig. 2E–G).

Similarly, flow cytometric analysis of secondary lymphoid tissue of *PI3Kδ* ^*D910A/D910A*^ recipients showed fewer CD4^+^ and CD8^+^ effector T cells as compared to WT recipients (*p < 0.05, *t*-test, *n* = 6/group) (Supplementary Fig. 3A, B, D, and E). However, there was significant decrease in the percentage of Tregs in secondary lymphoid tissue of *PI3Kδ* ^*D910A/D910A*^ recipients of BALB/c allografts compared to WT recipients at day 7 post-transplant (*p < 0.05, *t*-test, *n* = 6/group) (Fig. 2d and Supplementary Fig. 3C).

### PI3Kγ or PI3Kδ deletion and long-term allograft survival

**Fig. 3:**
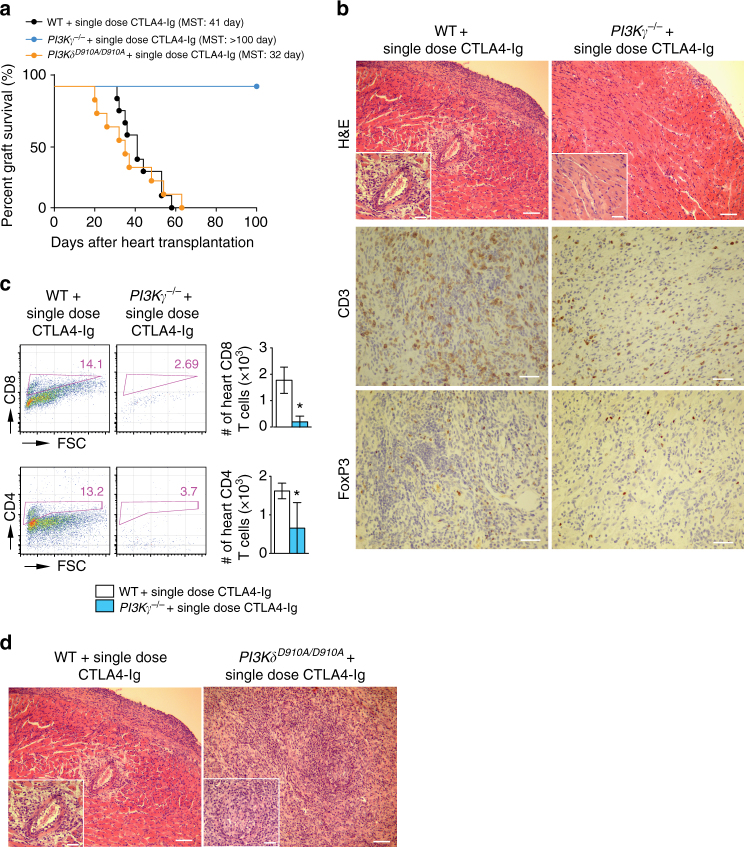
PI3Kγ or PI3Kδ deletion and long-term allograft survival. **a** single dose CTLA4-Ig, injected into *PI3Kγ* ^*−/−*^ recipients induced long-term acceptance of fully mismatched BALB/c cardiac allografts compared to the controls (MST of 41 vs. >100 days, *p < 0.05, *t*-test, *n* = 10/group). However, single dose CTLA4-Ig, injected into *PI3Kδ* ^*D910A/D910A*^ recipients of fully mismatched BALB/c cardiac allografts showed reduced allograft survival compared to the controls (MST: 41 vs. 32 days, *p < 0.05, *t*-test, *n* = 7–10/group). **b** Representative examples of cardiac allograft histology at day 100 post-transplant show lower grades of acute cellular rejection in the *PI3Kγ* ^*−/−*^ hearts treated with single dose CTLA4-Ig compared to cardiac allograft at day 28 post-transplant from WT mice treated with single dose CTLA4-Ig (*n* = 4/group). *Inset* show worse vasculopathy in the WT recipients treated with single dose CTLA4-Ig compared to *PI3Kγ* ^*−/−*^ hearts treated with single dose CTLA4-Ig (H&E stain, *scale bar* 50 μm, *inset scale bar* 25 μm). Immunohistochemistry analysis of CD3 and FoxP3 shows fewer CD3+ infiltrating cells in the *PI3Kγ* ^*−/−*^ hearts treated with single dose CTLA4-Ig compared to WT but relatively more FoxP3 (*scale bar* 50 μm). **c** Representative dot plots of leukocytes isolated from the allograft of *PI3Kγ* ^*−/−*^ and WT recipients treated with single dose CTLA4-Ig show significantly less CD4^+^ and CD8^+^ infiltration in the *PI3Kγ* ^*−/−*^ allografts compared to WT. *Bar graph* represents the absolute count of CD4^+^ and CD8^+^ T cells in the heart allografts of *PI3Kγ* ^*−/−*^ and WT recipients treated with single dose CTLA4-Ig. (*p < 0.05, *t*-test, *n* = 6/group, the graphs show data as mean ± s.e.m.). **d** Representative examples of cardiac allograft histology at day 28 post-transplant show higher grades of acute cellular rejection in the *PI3Kδ* ^*D910A/D910A*^ hearts treated with single dose CTLA4-Ig compared to WT mice treated with single dose CTLA4-Ig (*n* = 4/group). *Inset* show more severe vasculitis in the *PI3Kδ* ^*D910A/D910A*^ treated with single dose CTLA4-Ig compared to WT-recipient hearts treated with single dose CTLA4-Ig (H&E stain, *scale bar* 50 μm, inset *scale bar* 25 μm). (MST mean survival time)

While single dose CTLA4-Ig treatment (250 µg on day 2) of WT recipients resulted in moderate prolongation of allograft survival compared to untreated WT control (MST of 41 vs. 7 days, respectively, *p < 0.05, *t*-test), *PI3Kγ* ^*−/−*^ recipients treated with a single dose CTLA4-Ig showed indefinite prolongation of heart allograft survival (MST of >100 vs. 41 days, respectively, *p < 0.05, *t*-test, *n* = 7–10/group) (Fig. 3a). However, *PI3Kδ* ^*D910A/D910A*^ recipients treated with single dose CTLA4-Ig showed relatively shorter heart allograft survival compared to WT recipients (MST of 32 vs. 41 days, respectively, *p < 0.05, *t*-test, *n* = 7–10/group) (Fig. 3a). Histological examination of heart allografts (harvested at day 100 post-transplant) showed minimal signs of rejection in the *PI3Kγ* ^*−/−*^ + single dose CTLA4-Ig group as compared to WT + single dose CTLA4-Ig group (heart allografts harvested at day 28 post-transplant) (Fig. 3b). *PI3Kγ* ^*−/−*^+single dose CTLA4-Ig recipients also showed fewer CD4^+^ and CD8^+^ T cells in heart grafts harvested at day 28 post-transplant compared to WT + single dose CTLA4-Ig group. (*p < 0.05, *t*-test, *n* = 6/group) (Fig. 3c). However, histological examination of heart allografts harvested at day 28 post-transplant showed massive cell infiltration in the *PI3Kδ* ^*D910A/D910A*^ recipients treated with single dose CTLA4-Ig as compared to WT recipients treated with single dose CTLA4-Ig (Fig. 3d).

*PI3Kγ* ^*−/−*^ + single dose CTLA4-Ig recipients also showed significant suppression of CD4^+^ and CD8^+^ effector T cells in the secondary lymphoid tissue as compared to control mice (Supplementary Fig. 4A, B). A significant reduction in T cell proliferative responses and in the level of inflammatory cytokines IFNγ and interleukin (IL)-6 were also noted in *PI3Kγ* ^*−/−*^ + single dose CTLA4-Ig recipients (Supplementary Fig. 4C–E). These data indicate that the inhibition of PI3Kγ selectively suppresses alloreactive T cells.

### PI3Kδ deletion abrogates the tolerogenic effect of CTLA4-Ig

**Fig. 4:**
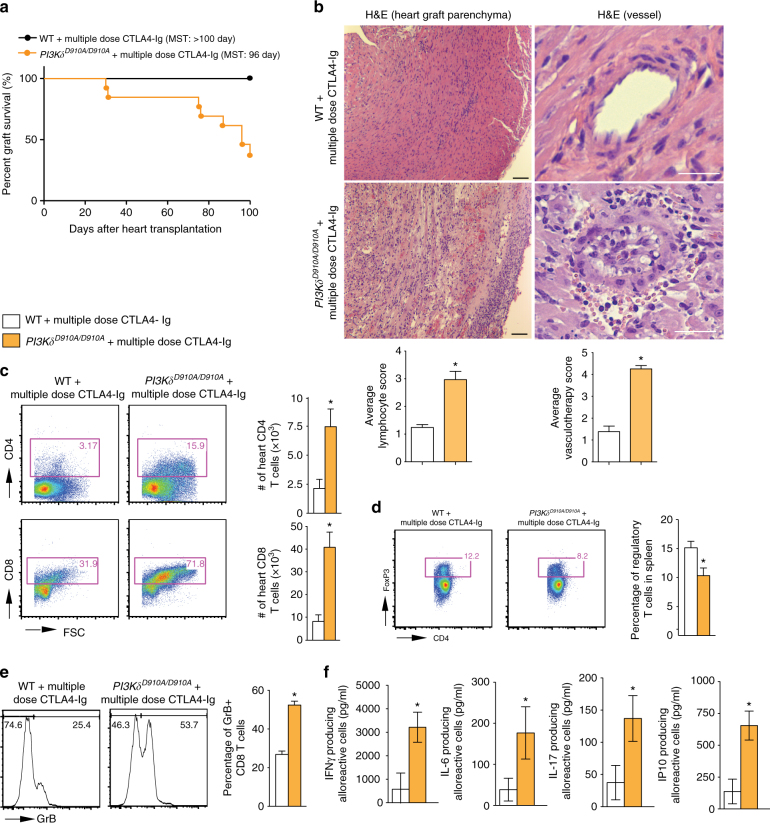
PI3Kδ deletion abrogates the tolerogenic effect of CTLA4-Ig. **a** Multiple dose CTLA4-Ig treatment of *PI3Kδ* ^*D910A/D910A*^ recipients abrogates the long-term acceptance of fully mismatched BALB/c cardiac allografts seen in treated WT controls (MST of > 100 vs. 96 days, *p < 0.05, *t*-test, *n* = 10/group). **b** Representative examples of cardiac allograft histology harvested 100 days after transplant show higher grades of cellular infiltration (H&E stain, *scale bar* 50 μm) and vasculopathy (H&E stain, *scale bar* 25 μm) in the *PI3Kδ* ^*D910A/D910A*^ hearts treated with multiple dose CTLA4-Ig compared to WT mice treated with multiple dose CTLA4-Ig (*n* = 4/group). The bar graphs show the average of lymphocyte infiltration score and vasculopathy score. The *PI3Kδ* ^*D910A/D910A*^ hearts treated with multiple dose CTLA4-Ig shows significantly higher score compared to WT mice treated with multiple dose CTLA4-Ig (*p < 0.05, *t*-test, *n* = 4/group, the graphs show data as mean ± s.e.m.). **c** Representative examples of dot plots of leukocytes isolated from the allograft of *PI3Kδ* ^*D910A/D910A*^ and WT recipients treated with multiple dose CTLA4-Ig shows more CD4^+^ and CD8^+^ infiltration in the allografts of *PI3Kδ* ^*D910A/D910A*^ recipients compared to WT control. Graph represents the absolute count of graft-infiltrating CD4 and CD8 in *PI3Kδ* ^*D910A/D910A*^ recipients treated with multiple dose CTLA4-Ig compared to WT control treated with multiple dose CTLA4-Ig (*p < 0.05, *t*-test, *n* = 6/group). **d** Representative examples of *dot plots* of Tregs in the DLN of *PI3Kδ* ^*D910A/D910A*^ and WT recipients treated with multiple dose CTLA4-Ig show significantly fewer Tregs in the *PI3Kδ* ^*D910A/D910A*^ recipients compared to WT control (*p < 0.05, *t*-test, *n* = 6/group). **e** Representative examples of histograms of GrB expression by CD8^+^ CD44^high^ T cells in the spleen of *PI3Kδ* ^*D910A/D910A*^ and WT recipients treated with multiple dose CTLA4-Ig show significantly more GrB expression in CD8^+^ of the *PI3Kδ* ^*D910A/D910A*^ recipients compared to WT (*p < 0.05, *t*-test, *n* = 4/group). **f** WT and *PI3Kδ* ^*D910A/D910A*^ recipient splenocytes were stimulated with donor splenocytes. Bar graphs represent the level of IFNγ, IL-6, IL-17, and IP10 in the supernatant collected from the MLR assay as measured by luminex (*p < 0.05, *t*-test, *n* = 4/group). **c**–**f** The graphs show data as mean ± s.e.m. (MST mean survival time; DLN draining lymph node; Treg regulatory T cell; GrB GranzymeB)

We then escalated the dose of CTLA4-Ig to induce indefinite allograft survival in WT recipients. Multiple dose CTLA4-Ig treatment (500 µg on day 0 then 250 µg on day 2, 4, 6, 8, and 10) of WT recipients resulted in indefinite prolongation of heart allograft survival compared to the untreated WT control (MST of >100 vs. 7 days, respectively, *p < 0.05, *t*-test). However, *PI3Kδ* ^*D910A/D910A*^ + multiple dose CTLA4-Ig group showed significantly earlier rejection as compared to WT recipients (MST of >100 vs. 96 days, respectively, *p < 0.05, *t*-test, *n* = 10/group) (Fig. 4a). Heart allografts from *PI3Kδ* ^*D910A/D910A*^ + multiple dose CTLA4-Ig treated mice recovered at day 100 post-transplant showed much more severe chronic rejection with lymphocyte infiltration and vasculopathy (Fig. 4b). Average scores for lymphocyte infiltration and vasculopathy were higher in *PI3Kδ* ^*D910A/D910A*^ + multiple dose CTLA4-Ig group compared to control (Fig. 4b below the H&E pictures) with increased intima-media thickness (Supplementary Fig. 5). *PI3Kδ* ^*D910A/D910A*^ + multiple dose CTLA4-Ig group showed significantly greater CD4^+^ and CD8^+^ infiltration of the allografts compared to WT + multiple dose CTLA4-Ig group (*p < 0.05, *t*-test, *n* = 6/group) (Fig. 4c). Furthermore, a significantly lower percentage of Tregs was observed in the spleen of treated *PI3Kδ* ^*D910A/D910A*^ mice compared to treated WT (*p < 0.05, *t*-test, *n* = 6/group) (Fig. 4d). A much higher number of CD8^+^ effector T cells were also observed in the secondary lymphoid organs of treated *PI3Kδ* ^*D910A/D910A*^ mice compared to treated WT (*p < 0.05, *t*-test, *n* = 6/group) (Supplementary Fig. 6). Interestingly, splenic CD8^+^ CD44^high^ T cells from *PI3Kδ* ^*D910A/D910A*^ mice showed much higher expression of Granzyme B (GrB) as compared to WT recipients, as measured by flow cytometry (52.38% ± 2.0% vs. 26.83% ± 1.77%, respectively, *p < 0.05, *t*-test, *n* = 4/group) (Fig. 4e). Finally, *PI3Kδ* ^*D910A/D910A*^ recipient splenocytes also significantly increased inflammatory cytokine and chemokine production upon stimulation by irradiated donor cells, as measured by luminex assay (*p < 0.05, *t*-test, *n* = 4/group) (Fig. 4f).

### PI3Kδ deletion reduces Treg survival

**Fig. 5:**
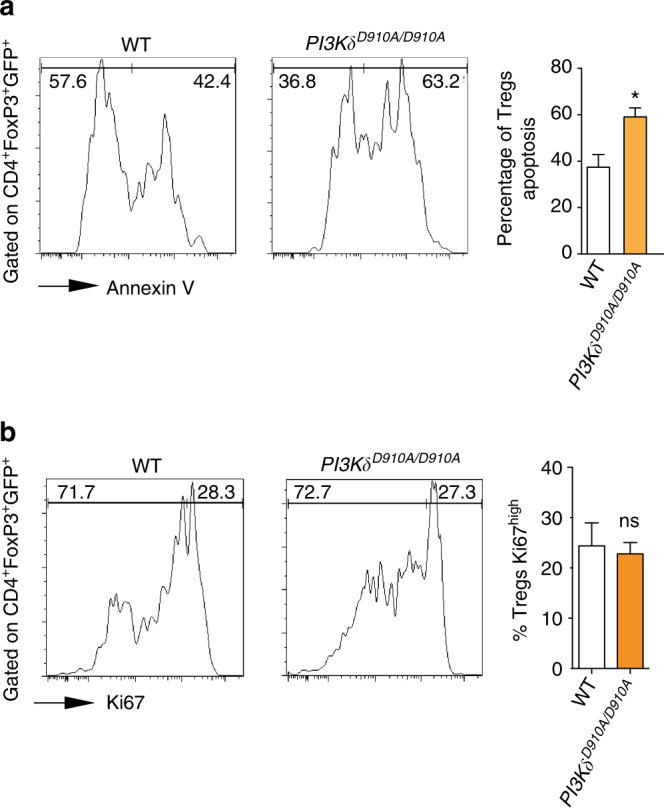
PI3Kδ inhibition abrogates Treg survival. **a** Representative examples of Annexin staining of WT and *PI3Kδ* ^*D910A/D910A*^ nTregs transferred in the spleen of C57BL/6 mice induced with GVHD. Bar graph represents the different percentages. (*p < 0.05, *t*-test, *n* = 3/group). **b** Representative examples of Ki67 expression of WT and *PI3Kδ* ^*D910A/D910A*^ nTregs transferred into C57BL/6 mice induced with GVHD. Bar graph represents the different percentages. (*p < 0.05, *t*-test, *n* = 3/group). **a**, **b** The graphs show data as mean ± s.e.m. (Treg regulatory T cell; GVHD graft vs. host disease; nTreg natural regulatory T cell)

Two groups of lethally irradiated BALB/c mice were reconstituted with 2 × 10^6^ CD3^+^CD25^−^ T cells derived from WT C57BL/6 (Thy1.1) mice. Mice were co-injected with 1 × 10^6^ WT and *PI3Kδ* ^*D910A/D910A*^ natural regulatory T cells (nTreg) (Thy1.2). As shown in Fig. 5a, flow cytometric analysis of splenocytes showed that *PI3Kδ* ^*D910A/D910A*^ nTregs had higher rate of apoptosis compared to WT nTregs (*p < 0.05, *t*-test, *n* = 3/group). No difference was seen in the proliferation of the transferred WT and *PI3Kδ* ^*D910A/D910A*^ nTregs as measured by Ki67 using flow cytometry. (*p* = ns, *t*-test, *n* = 3/group) (Fig. 5b).

### PI3Kγ deletion protects from chronic rejection

Chronic rejection remains a major cause of graft failure in clinical solid organ transplants^25, 26^. We next investigated the role of PI3Kγ inhibition in chronic rejection using class II MHC-mismatch heart transplant model (B6.H-2^bm12^ donor hearts into C57BL/6 recipient). Heart allografts recovered from *PI3Kγ* ^*−/−*^ recipients day 28 showed significantly less infiltration, fibrosis and intima-medial thickness as compared to WT recipients (Supplementary Fig. 7).

### PI3Kδ deletion has no effect on *FoxP3* methylation

We assessed the expression of the genes controlling the *FoxP3* promoters and the status of *FoxP3* demethylation on multiple CpG motifs of the first intron of *FoxP3*, which have been shown to control its transcription^27, 28^. We performed quantification analysis of the *FoxP3* gene methylation at the five CpG sites of the proximal promoter known to control its transcription (location: −6750 to −6714 from *ATG*) in WT and *PI3Kδ* ^*D910A/D910A*^ Tregs at 24 h post stimulation. Our analysis did not show any statistically significant difference between the different groups (Supplementary Fig. 8). Total percent methylation for in WT and *PI3Kδ* ^*D910A/D910A*^ were 58.3 ± 6.6 and 59.29 ± 11.2, respectively, (*p* = 0.8, *t*-test, *n* = 4/group).

### Generation of PI3Kδ and PI3Kγδ selective inhibitors

**Fig. 6:**
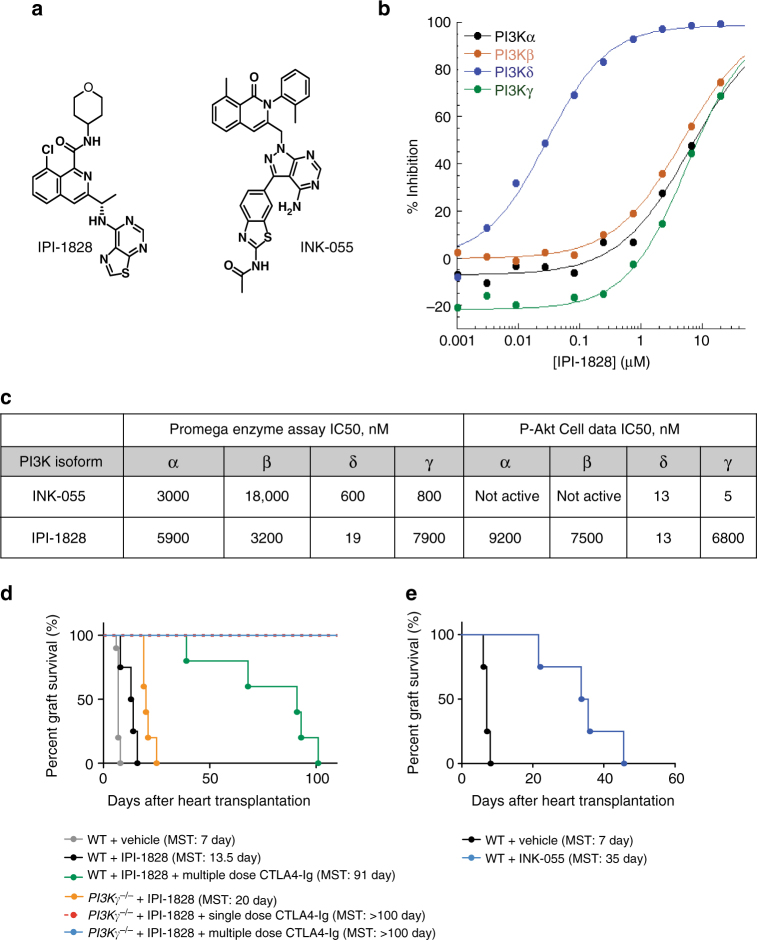
Synthesis of new PI3K δ and γδ inhibitor. The characterization of IPI-1828, a PI3Kδ selective compound, and INK-055, a PI3Kγδ inhibitor. **a** The chemical structure of IPI-1828 and INK-055. **b** Isotherms of IPI-1828 inhibitory activity in enzymatic assays for Class 1 PI3Ks were determined by monitoring the hydrolysis of ATP with a luminescence-based end point assay as described in methods section. This assay was run in the presence of 3 mM ATP and 500 uM PIP_2_ substrates. The ATP concentration of 3 mM was used to more to closely resemble cellular concentrations. **c** Summary of IC50s in a biochemical assay run in the presence of 3 mM ATP, and isoform selective cell-based assays for class 1 PI3Ks. **d** Synergism of PI3Kδ and PI3Kγ inhibition in prolonging allograft survival. Kaplan–Meier survival of heart allografts in WT and *PI3Kγ* ^*−/−*^ recipients treated with PI3Kδ inhibitor (IPI-1828) and single dose CTLA4-Ig or multiple dose CTLA4-Ig. (*n* = 5/group). **e** Kaplan–Meier survival of heart allograft in WT recipients treated with PI3Kγδ inhibitor (INK-055) showed significant prolongation of heart allograft survival compared to control (MST of 35 vs. 7 days, *p < 0.05, *t*-test, *n* = 5/group). (MST mean survival time)

To examine the effect of pharmacologic inhibition of PI3K isoforms, the selective inhibitors IPI-1828 and INK-055 were synthesized and utilized in our transplant model. To mimic the high ATP concentrations found within cells, IPI-1828 and INK-055 were evaluated in PI3K isoform specific biochemical assays in the presence of 3 mM ATP. IPI-1828 is a PI3Kδ selective inhibitor with activity against the targeted isoform of 19 nM against PI3Kδ and 5900, 3200, and 7900 nM against PI3Kα, PI3Kβ, and PI3Kγ, respectively. IPI-1828 was evaluated in isoform selective cell based assays with a 13 nM IC50 of the phosphorylation of AKT (S473) in the PI3Kδ selective assay, and greater than 200-fold less activity in the PI3Kα, PI3Kβ, and PI3Kγ isoform selective cell based assays. INK-055 is a previously characterized PI3K-inhibitor that has potent cellular activity against PI3Kδ and PI3Kγ with IC50s of 13 and 5 nM, respectively, (Fig. 6a–c) and is a dual PI3Kγδ inhibitor in animal models^29^.

### Synergistic effect of PI3Kγ and PI3Kδ inhibition

We then tested whether the presence of PI3Kγ inhibition would rescue the deleterious effect of PI3Kδ inhibition and whether there is synergism in further prolonging heart allograft survival. Naive BALB/c allografts were transplanted into WT C57BL/6 recipients and treated with a specific PI3Kδ inhibitor (IPI-1828: 50 mg/kg twice daily via gavage day 0 to 6). As shown in Fig. 6d, WT recipients treated with PI3Kδ inhibitor (IPI-1828) exhibited prolonged allograft survival compared to the WT recipients treated with vehicle only (MST of 13.5 vs. 7 days, respectively, *p < 0.05, *t*-test, *n* = 5/group). However, mice treated with dual PI3Kγδ inhibitor (INK-055: 100 mg/kg daily, day 0 to 6) exhibited an additional allograft survival compared to either the WT recipients treated with PI3Kδ inhibitor (IPI-1828) or vehicle only (MST of 35 vs. 13.5 vs. 7 days, respectively, *p < 0.05, *t*-test, *n* = 5/group) (Fig. 6d, e). Furthermore, *PI3Kγ* ^*–/–*^ recipients treated with PI3Kδ inhibitor (IPI-1828) exhibited a significantly prolonged allograft survival compared to the *PI3Kγ* ^*–/–*^ recipients treated with vehicle only (MST of 20 vs. 11 days, respectively, *p < 0.05, *t*-test, *n* = 5–10/group) (Fig. 6d). Interestingly, while PI3Kδ inhibitor (IPI-1828) abrogated the tolerogenic effect of multiple dose CTLA4-Ig on WT C57BL/6 recipients of BALB/c hearts (MST of 91 days), adding PI3Kδ inhibitor (IPI-1828) to *PI3Kγ* ^*–/–*^ recipients treated with single or multiple dose CTLA4-Ig induced indefinite heart allograft survival and did not abrogate tolerance (Fig. 6d).

## Discussion

To study the role of PI3Kγ and PI3Kδ in alloimmunity, first we showed that *PI3Kγ* ^*−/−*^ and *PI3Kδ* ^*D910A/D910A*^ T cells have impaired alloimmune reactivity; demonstrating suppressed alloreactive T cell proliferative responses coupled with a reduction in the frequency of IFNγ producing alloreactive cells in vitro. These findings were confirmed in vivo in an adoptive transfer model. These data are in accordance with previously published work showing that, along with activation via G protein coupled receptors, TCR cross-linking with anti-CD3 Abs also activates p110γ and induces its association with lck and ZAP70, resulting in T cell activation^14^. PI3Kδ is also downstream of TCR and its absence from this pathway explains the T cell defect observed in vitro and in vivo. However, PI3Kδ deletion had a significant deleterious effect on regulatory T cell (Treg) induction.

Prolongation of heart allograft survival in *PI3Kγ* ^*−/−*^ recipients was associated with a marked suppression of alloreactive T cells in a model of acute heart allograft rejection. Accumulating experimental data point to the importance of targeting innate immunity and inflammation to improve transplant outcomes, particularly to prevent the development of chronic rejection, which remains one of the main barriers to long term allograft survival^30–35^. *PI3Kγ* ^*−/−*^ recipients showed a notable decrease in inflammatory cytokines known to play a key role in the pathogenesis of chronic rejection^36, 37^. This reduction in inflammatory cytokines may, in part, explain the marked reduction in chronic rejection noted in the *PI3Kγ* ^*−/−*^ recipients.

One of our key findings was that the inhibition of PI3Kδ effectively suppressed alloreactive T effectors while it also reduced Tregs. The balance of Tregs to T effectors is well established as a key determinant of the long-term outcome of transplantation. Similar to PI3Kγ inhibition, PI3Kδ inhibition prolonged allograft survival in an acute heart allograft rejection model with significant suppression of effector CD4^+^ and CD8^+^ T cells post-transplantation. However, to study the impact of PI3Kδ signaling on the maintenance of long-term tolerance via its effect on Treg homeostasis, we used a combinatorial strategy, treating *PI3Kδ* ^*D910A/D910A*^ recipients with single dose CTLA4-Ig. In this long-term transplantation model, graft survival is dependent on the presence of Tregs. PI3Kδ inhibition abrogated the tolerogenic effect of CTLA4-Ig with a significant reduction in Tregs. This decrease in Tregs may explain the upregulation of the CD8^+^ T effector cells in *PI3Kδ* ^*D910A/D910A*^ recipients and the observed increase in allograft-infiltrating inflammatory cells.

We then investigated whether this reduction in Tregs in the periphery is due to decreased proliferation or increased apoptosis. Transfer of natural Tregs (nTreg) from WT, and *PI3Kδ* ^*D910A/D910A*^ mice into an allogeneic host showed a higher rate of *PI3Kδ* ^*D910A/D910A*^ nTreg apoptosis compared to WT nTregs. No difference in proliferation was observed between the different groups, as assessed by Ki67 expression of transferred Tregs. Our data is in accordance with previous work by Ali et al.^38^, where p110δ inhibition induced a Treg defect, leading to tumor regression through enhanced CD8 cytotoxic T cell activity.

We also investigated the mechanisms responsible for the deleterious effect of PI3Kδ on Treg induction. The transcriptional activity of the CpG-motif containing element of the *FoxP3* first intron, which is located within TSDR (Treg-specific demethylated region) is known to be methylation-sensitive. Methylation of this island inversely correlates with CREB binding and *FoxP3* expression^28^. Furthermore, DNA methylation in TSDR has been shown to be critically involved in maintaining stable *FoxP3* expression^39^. We observed no differences in the demethylation of the *FoxP3* gene in *PI3Kδ* ^*D910A/D910A*^ CD4^+^ Tregs.

Currently, the importance of PI3K pathways in the development of Tregs is under intense investigation^40^, and dramatic anti-tumor effects have been seen in trials using PI3Kδ inhibition in patients with chronic lymphocytic leukemia^41^. This effect has been ascribed to the inhibition of Treg in these patients and our data are in concordance with these findings.

Altogether, our data indicate that the inhibition of PI3Kγ and PI3Kδ both restrict the expansion of alloreactive T cells, however, PI3Kδ has a counter-regulatory effect on the homeostasis of Treg. Recent studies have highlighted the potential importance of the level of AKT (activated by the PI3K pathway) on the fate of Treg and differentiation of effector T cells^42, 43^. Modulation of AKT activity in Treg is essential for their optimal function and excessive AKT activation has been shown to be detrimental to their suppressive activity^40, 44, 45^.

On the other hand, upon TCR activation, rapidly dividing CD8^+^ cells demonstrate high AKT activity levels. Effector T cells with high PI3K/AKT activity are resistant to the suppressive effects of Tregs^46, 47^. Using mice deficient for PTEN (natural inhibitor of PI3K) in Treg, Turka’s group has shown a change in the metabolic programming of Treg and decline in their stability^48^. Future studies are required to better define the relative contribution of PI3Kδ on controlling the balance of Teff/Treg via regulation of metabolic programming.

Finally, we have generated a PI3Kδ inhibitor (IPI-1828) and a dual PI3Kγδ inhibitor (INK-055). PI3Kδ inhibition has already entered the clinic with success, though synthesizing a PI3Kγ inhibitor has proved a more daunting task. In our studies, both PI3Kγ and PI3Kδ inhibition suppressed alloreactive T cells; therefore, an important question to address was the importance of combinatorial therapy in prolonging heart allograft survival. Dual inhibition showed a further suppression of alloimmune responses and restored the tolerogenic effect of CTLA4-Ig in the presence of PI3Kδ inhibition. Dual inhibition of PI3Kγ and δ has also previously been shown to have superior anti-inflammatory effects^10^. Our data indicate that PI3Kγ inhibition rescues the negative effect of PI3Kδ inhibition on long-term allograft survival and identifies dual PI3Kγ and δ inhibition as a promising and novel platform to pursue in future studies with large animals.

A role for PI3Kγ inhibition in modifying the activity of tumor-infiltrating myeloid cells toward a pro-inflammatory phenotype, leading to increased anti-tumor activity and reduced tumor growth has recently been reported^49, 50^. The contrasting findings observed here could be due to differences between our allogeneic transplant model versus syngeneic tumor models. In heart transplant models, rejection is predominantly a T cell mediated process^51^. Myeloid cells do not constitute the majority of infiltrating cells during rejection. Our T cell data is in accordance with multiple other publications showing a direct role for PI3Kγ inhibition in T cell suppression^12–14, 52–54^. Chronically immunosuppressed patients suffer from increased risk of malignancy and the capacity of PI3Kγ to potentially both prevent rejection and have anti-tumor activity renders it an extremely attractive target for clinical translation.

The field of transplantation has an ongoing unmet need for more selective and effective immunomodulatory molecules to improve transplantation outcomes. PI3Kγδ inhibitors are being clinically studied for other immune-mediated diseases and our data indicate that these molecules also hold significant promise in controlling alloimmune activation, and show particularly impressive effects in the prevention of chronic allograft rejection, a major cause of late allograft loss. Further studies are required to fully evaluate their potential role in clinical transplantation.

## Methods

### Mice and reagents

C57BL/6J (JAX#000664), BALB/cByJ (H-2^d^) (JAX#001026), B6.C-H2^*bm12*^/KhEg (bm12) (JAX#001162), *Foxp3GFP* knock-in mice on a C57BL/6 background (JAX#023800) and *Rag* ^*−/−*^ mice (JAX#002216) were obtained from the Jackson Laboratory. *PI3Kγ* ^*−/−*^ C57BL/6 mice (backcrossed 11 generations) were received from Dr Bao Lu (Boston Children’s Hospital/ Harvard Medical School) and maintained in our animal facility^55^. p110^D910A^ C57BL/6 (*PI3Kδ* ^*D910A/D910A*^) mice were obtained from Charles River Laboratory^56^. *PI3Kγ* ^*−/−*^ C57BL/6 and p110^D910A^ C57BL/6 (*PI3Kδ* ^*D910A/D910A*^) mice were also backcrossed with *FoxP3GFP* knock-in mice in our facility. Male or female mice were used at 6–10 weeks of age (20–25 g) and were housed in sterilized ventilated cages in a specific pathogen-free animal facility under standard 12 h light/12 h dark cycle. Mice were fed ad libitum irradiated food and water. Each individual experiments was done using three to ten mice per group. Mice were euthanized by either CO_2_ inhalation, intraperitoneal injection of ketamine/xylazine or isoflurane inhalation followed by observation of loss of vital signs and cervical dislocation. All animal experiments and methods were performed in accordance with the relevant guidelines and regulations approved by the Institutional Animal Care and Use Committee of Brigham and Women’s Hospital, Harvard Medical School, Boston, MA.

### Murine cardiac transplantation

Vascularized intra-abdominal heterotopic transplantation of cardiac allografts was performed using microsurgical techniques as described previously^57^. Briefly, 1 ml of cold heparin (BD Vacutainer Sodium Heparin #366480 143USP units/10 ml) was infused into the inferior vena cava of the donor mouse. Hearts were harvested following the ligation/dissection of superior vena cava and inferior vena cava and dissection of ascending aorta and pulmonary artery. Harvested donor hearts were stored at 4 degrees celsius, immersed in UW (University of Wisconsin) solution until transplantation. After abdominal incision of recipient mouse, abdominal aorta and inferior vena cava were clamped. Ascending aorta and pulmonary artery of donor heart were attached to abdominal aorta and inferior vena cava of recipient mouse, respectively, using 10–0 suture. Beating of transplanted heart was observed upon removal of cross clamp, and abdominal incision was closed. The survival of cardiac allografts was assessed by daily palpation. Rejection was defined as complete cessation of cardiac contractility as determined by direct visualization and confirmed by histology.

### Skin transplantation

Full-thickness trunk skin grafts (1 cm^2^) harvested from BALB/c donors were transplanted onto the flank of *Rag* ^*−/−*^-recipient mice, sutured with 6–0 silk, and secured with dry gauze and a bandage for 7 days.

### Histological and immunohistochemical assessment

Five micron thick formalin fixed paraffin embedded sections were stained with standard Hematoxylin and Eosin stain (H&E), CD3 stain, Foxp3 stain, Elastic Van Gieson stain and F4/80 stain. Histological evaluation was done using a score modified from the International Society for Heart and Lung Transplantation^58, 59^. Lymphocyte infiltration is graded 0 to 4 at 6 random fields of each heart H&E section blindly by two individual researchers (six sections/heart, three mice per group). The grades are defined as follows: grade 0 (no lymphocyte infiltration), grade 1 (less than 25% lymphocyte infiltration), grade 2 (25 to 50% lymphocyte infiltration), grade 3 (50 to 75% lymphocyte infiltration), and grade 4 (more than 75% lymphocyte infiltration and myocyte hemorrhage). Vascular score is determined by a combination of vascular occlusion score and perivascular lymphocyte infiltration. Vascular occlusion is scored from grade 0 to 4 for every vessel (six sections/heart, three mice per group). The grades are defined as follows: grade 0 (no occlusion), grade 1 (less than 50% occlusion), grade 2 (50 to 75% occlusion), grade 3 (75 to 95% occlusion) and grade 4 (more than 95% occlusion). The perivascular lymphocyte infiltration is scored as described above and was then added to the vascular occlusion score.

### Isolation of lymphocytes from hearts

Cardiac allografts were removed, perfused with PBS, minced finely with a razor blade and digested at 37 °C with 1 mg/ml collagenase in 1 ml complete medium for 1 h. Cells in the supernatant were washed twice, centrifuged at 620x*g* using Percoll solutions at 33% (cell suspension) and 66%. Lymphocytes were aspirated at the interface.

### Treg generation assay

Using a 96-well flat bottom plate, 100 μl of anti-CD3 antibody (Ab) diluted in PBS (1 μg/ml, BD Biosciences) was added to each well and incubated at 37 °C for 4 h. In all, 1 μg/ml of anti-CD28 Ab, 10 ng/ml of IL-2 and soluble recombinant human TGFβ (1 ng/ml, R&D Systems) was then added to each well in the presence of 2.5 × 10^4^ CD4^+^CD25^−^ T cells of C57BL/6 or *PI3Kγ* ^*−/−*^ mice.

### CD3/CD28 T cell stimulation and MLR assays

Using a 96-well flat bottom plate, 100 μl of anti-CD3 Ab and soluble anti-CD28 Ab (1 μg/ml, BD Biosciences) was used in the presence of C57BL/6 or *PI3Kγ* ^*−/−*^ splenocytes. For the MLR assay, irradiated wild type (WT) BALB/c splenocyte stimulators and WT C57BL/6 or *PI3Kγ* ^*−/−*^ splenocyte responders were added to each well in a 96-well round bottom plate pulsed with 1 μCi of tritiated thymidine (^3^H) and the incorporation efficiency was determined.

### ELISpot

ELISpot assays were performed with WT BALB/c splenocytes as stimulators and WT C57BL/6 or *PI3Kγ* ^*−/−*^ splenocytes as responders, as described previously^60^. Briefly, 5 × 10^5^ each of irradiated stimulating cells and responders were added to each well of Millipore Immunospot plates (Millipore Corporation, Bedford, MA). Biotinylated antibodies specific for each cytokine were added to the wells and incubated. The plates were developed and spots were counted on an Immunospot analyzer (Cellular Technology Ltd., Cleveland, OH).

### Flow cytometric analysis

Anti-mouse monoclonal antibodies against CD62L (1:400 diluted, MEL-14, 560516), CD44 (1:400 diluted, IM7, 103027), CD4 (1:400 diluted, RM4-5, 100559), CD25 (1:400 diluted, PC61.5, 12-0251-81B), CD8 (1:400 diluted, 53-6.7, 100734), FoxP3 (1:300 diluted, FJK-16s, 11-5773-82), GrB (1:100 diluted, KGZB, 12-8898-82), H2K^b^ (1:100 diluted, SF1-1.1, 553565), thy1.2 (1:100 diluted, 53-2.1, 17-0902-83), Annexin PE (51-65875X), 7 AAD PerCP (51-68981E), Ki67 (1:300 diluted, SolA15, 11-5698-82), cRel (1:300 diluted, IRELAHS, 12-6111-80) were purchased from BD Biosciences, San Jose, CA. Cells recovered from spleens and peripheral lymphoid tissues were interrogated with a FACS Canto-II flow cytometer (BD Biosciences) and analyzed using FlowJo software version 9.3.2 (Treestar, Ashland, OR). Para-aortic lymph nodes from host were harvested as secondary lymphoid organs or draining lymph nodes (DLN) of the heart allografts. Gating strategies for lymphocyte subpopulations are shown in Supplementary Fig. 9.

### Luminex assay

A 21-plex cytokine-kit (Millipore, St Charles, MO) was used according to the manufacturer’s instructions to assess cytokine production in culture supernatant and in murine serum samples.

### CFSE labeling

Spleen and peripheral lymph nodes were recovered from C57BL/6 (WT) and *PI3Kγ* ^*−/−*^ mice, and a single cell suspension was prepared in HBSS. Red blood cells were lysed with ACK buffer and cells were suspended in Hanks’ Balanced Salt Solution (HBSS) at 1 × 10^7^ cells/ml for labeling with CFSE (Molecular Probes, Portland, OR). Briefly, cells were incubated with CFSE at a final concentration of 5 μM in serum-free HBSS at room temperature for 6 min The labeling reaction was then terminated by the addition of FCS (10% of the total volume). Cells were then washed twice in HBSS and used for in vitro experiments.

### *FoxP3* gene demethylation analysis

For DNA demethylation analysis, 500 ng of extracted genomic DNA was bisulfite treated and purified according to the manufacturer’s protocol. PCRs were performed using 1 μl of bisulfite treated DNA and 0.2 μM of each primer. PCR products were sequenced by Pyrosequencing on the PSQ96 HS System (Pyrosequencing, Qiagen) following the manufacturer’s instructions. The methylation status of each CpG site was determined individually as an artificial C/T SNP using QCpG software (Pyrosequencing, Qiagen). The methylation level at each CpG site was calculated as the percentage of the methylated alleles divided by the sum of all methylated and unmethylated alleles. The mean methylation level was calculated using methylation levels of all measured CpG sites within the targeted region of each gene. Each experiment included non-CpG cytosines as internal controls to detect incomplete bisulfite conversion of the input DNA. In addition, a series of unmethylated and methylated DNA are included as controls in each PCR. Furthermore, PCR bias testing was performed by mixing unmethylated control DNA with in vitro methylated DNA at different ratios (0, 5, 10, 25, 50, 75, and 100%), followed by bisulfite modification, PCR, and Pyrosequencing analysis. CPG sites analyzed for the *FoxP3* gene were CpG 64–68 of the proximal promoter.

### PI3K inhibitors

IPI-1828 (PI3Kδ inhibitor) was prepared according the methods in US Patent No. 8,901,133 Example 85 and was obtained from Infinity Pharmaceuticals, Inc. INK-055 (PI3Kγδ inhibitor) was prepared as described^29^ and was obtained from Infinity Pharmaceuticals, Inc. IPI-1828 was stored as a 10 mM stock in dimethyl sulfoxide (DMSO) at room temperature. All human PI3K isoforms were purchased from Millipore (Billerica, MA).

### Biochemical IC_50_ determinations

IC_50_ values for all PI3K isoforms were determined by measuring the dose-dependent decrease in luminescent signal with increasing concentrations of IPI-1828. PI3K was incubated for 15 min with increasing concentrations of IPI-1828 followed by addition of ATP to 3 mM concentration and 500 μM diC_8_PIP_2_ substrates. Reactions were incubated at room temperature for 2 h. Detection of PI3K activity was then carried out using the ADP-Glo Max Assay Kit. Enzyme concentrations of 10 nM were used for the PI3Kα and PI3Kδ isoforms, and 40 nM for PI3Kβ and PI3Kγ isoforms. IC_50_ measurements were calculated as described previously^61^.

### PI3Kα assay design/PI3Κβ assay design

SKOV-3 cells (ATCC HTB-77) for PI3Kα assay design and 786-O cells for PI3Kβ assay design were seeded into 96-well cell culture grade plates at a density of 200,000 cells/200 μl/well of RPMI-1640 with 10% FBS. Cells were incubated overnight at 5% CO_2_ and 37 °C. IPI-1828 or INK-055 (diluted in 25% DMSO in threefold dilutions from 10 μM to generate an 11 and 9 point titration curve, respectively) was added to the cells, resulting in a final DMSO concentration of 0.5%, and incubated for 30 min at 5% CO_2_ and 37 °C.

### PI3Kγ assay design

RAW 264.7 cells were seeded into 96-well cell culture-grade plates at a density of 200,000 cells/200 μl/well of DMEM with no FBS added. Cells were incubated overnight at 5% CO_2_ and 37 °C. Following 18 h of serum-starvation, IPI-1828 or INK-055 (diluted in 25% DMSO in threefold dilutions from 0.2 μM to generate a 9-point titration curve) was added to the cells, resulting in a final DMSO concentration of 0.5%, and incubated for 30 min at 5% CO_2_ and 37 °C. Cells were then stimulated with 25 nM C5a for 3 min in the presence of IPI-1828 or INK-055.

### PI3Kδ assay design

RAJI cells were seeded into 96-well cell culture-grade plates at a density of 200,000 cells/200 μl/well of RPMI-1640 with no FBS added. Cells were incubated overnight at 5% CO_2_ and 37 °C. Following 18 to 24 h of serum-starvation, IPI-1828 or INK-055 (diluted in 25% DMSO in threefold dilutions from 10 μM to generate an 11-point titration curve) was added to the cells, resulting in a final DMSO concentration of 0.5%, and incubated for 30 min at 5% CO_2_ and 37 °C. Cells were then stimulated with 10 μg/ml anti-human IgM for 30 min in the presence of IPI-1828 or INK-055.

Media was aspirated and 50 μl/well of ice-cold lysis buffer was added. Plates were incubated on ice for 5 min and then centrifuged at 3000 rpm at 4 °C for 5 min Phospho AKT ELISA and analysis were as described for each isoform^61^.

### Pharmacokinetics

The pharmacokinetics and oral bioavailability of IPI-1828 in C57BL/6 mice were studied following a single intravenous and oral doses. Blood samples were collected prior to dose administration (oral dose only) and at time points post-treatment. The blood samples were processed to plasma and assayed for IPI-1828 using a liquid chromatography method with tandem mass spectrometric (LC-MS/MS) detection. Pharmacokinetic parameters were determined using standard non-compartmental methods^61^.

### Statistics

Kaplan–Meier survival curves were constructed, and a log rank comparison of the groups was used to calculate *p*-values. The unpaired *t*-test was used for comparison of experimental groups examined by ELISpot, ELISA, luminex, flow cytometry, and MLR. Differences were considered to be significant for *p* < 0.05. Prism software was used for data analysis and drawing graphs (GraphPad Software, Inc., San Diego, CA). Data represent mean ± s.e.m.

### Data availability

All data generated or analyzed during this study are available from the author on reasonable request.

## Electronic supplementary material

Supplementary Information
