# Supplementary material for: Regulation of T cell alloimmunity by PI3Kγ and PI3Kδ
Source: Nat Commun. 2017 Oct 16;8:951. doi: 10.1038/s41467-017-00982-x (PMC5643371; doi:10.1038/s41467-017-00982-x)
Supplement: Supplementary file 1 — Supplementary Information [file 41467_2017_982_MOESM1_ESM.pdf]

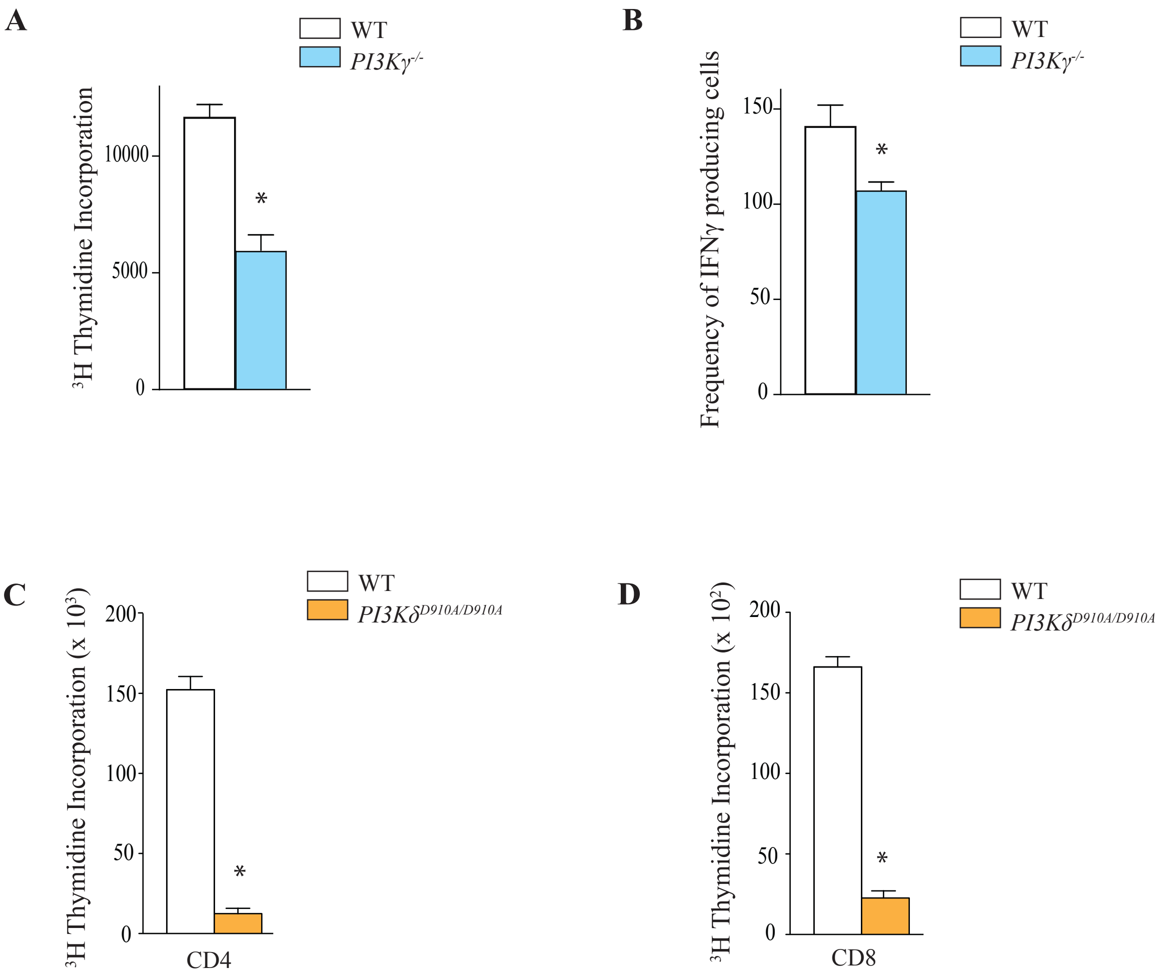

Supplementary Figure 1: PI3Kγ or PI3Kδ deletion impairs T cell activation *in vitro*: (A) *PI3Kγ*$^{-/-}$ splenocytes proliferated significantly less than WT T cells in response to allo-antigens as measured by thymidine incorporation. (B) *PI3Kγ*$^{-/-}$ splenocytes showed a lower frequency of IFNγ producing T cells when compared to WT T cells in an ELISpot assay with allo-antigens. (C) *PI3Kδ*$^{D910A/D910A}$ CD4^{+} T cells stimulated with anti-CD3/CD28 *in vitro*, proliferate less than WT T cells as measured by Thymidine incorporation. (D) Significantly lower proliferation of *PI3Kδ*$^{D910A/D910A}$ CD8^{+} T cells stimulated with anti-CD3/CD28 *in vitro*, as compared to WT T cells, as measured by Thymidine incorporation. (Data are representative of three separate experiments, *p<0.05, *t*-test, n=3-4 mice/group, the graphs show data as mean±s.e.m.).

Supplementary Figure 1

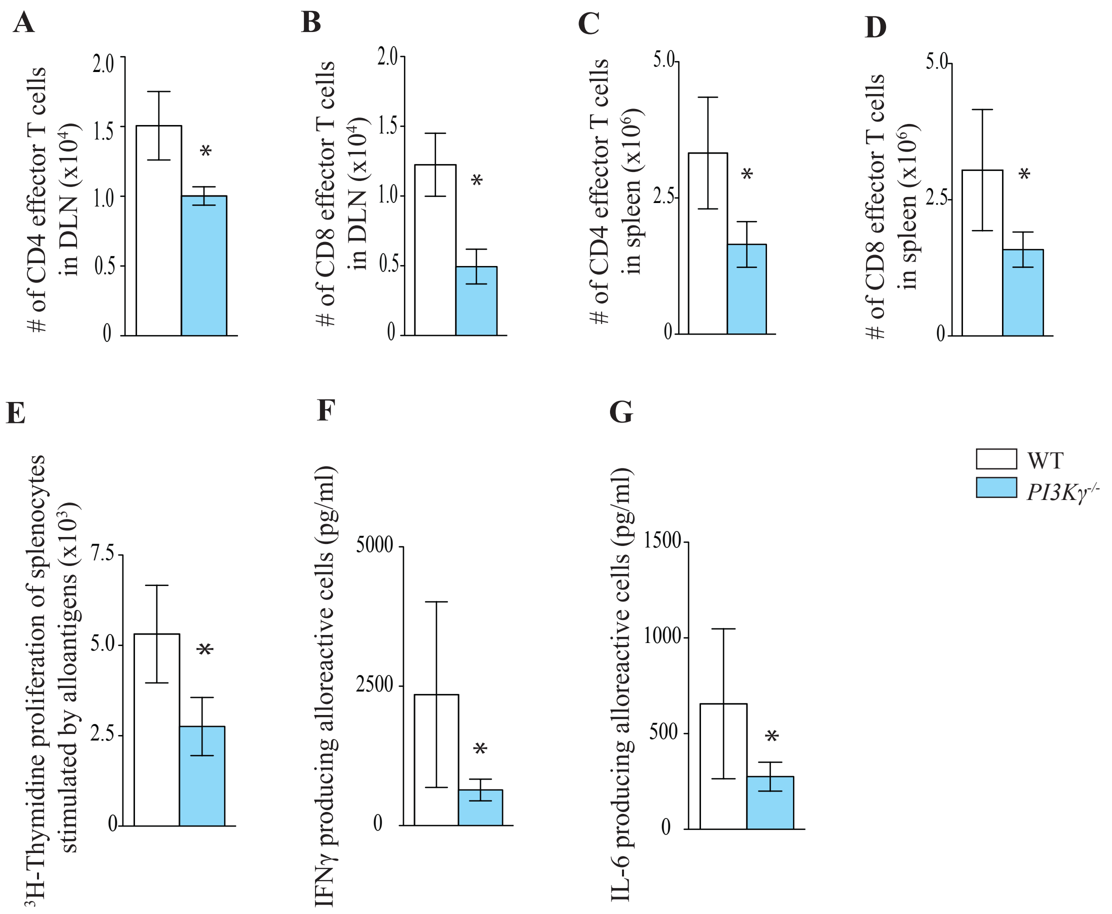

Supplementary Figure 2: PI3Kγ deletion suppresses Teff: Naïve BALB/c heart allografts were transplanted into fully allogeneic C57BL/6 (WT) or *PI3Kγ*$^{-/-}$ recipients. (A) Bar graph represents the absolute count of CD4^{+} Teff in DLN in *PI3Kγ*$^{-/-}$ recipients compared to WT at day 7 post-transplant (*p<0.05, *t*-test, n=6/group). (B) Bar graph represents the absolute count of CD8^{+} Teff in DLN in *PI3Kγ*$^{-/-}$ recipients compared to WT at day 7 post-transplant (*p<0.05, *t*-test, n=6/group). (C) Bar graph represents the absolute count of CD4^{+} Teff in splenocytes of *PI3Kγ*$^{-/-}$ recipients compared to WT at day 7 post-transplant (*p<0.05, *t*-test, n=6/group). (D) Bar graph represents the absolute count of CD8^{+} Teff in splenocytes of *PI3Kγ*$^{-/-}$ recipients compared to WT at day 7 post-transplant (*p<0.05, *t*-test, n=6/group). (E) Bar graph shows that *PI3Kγ*$^{-/-}$ recipient splenocytes proliferated less upon stimulation with donor splenocytes compared to WT. (F) Bar graph represents the level of IFNγ in the supernatant collected from the MLR assay as measured by luminex. (G) Bar graph represents the level of IL-6 in the supernatant collected from the MLR assay as measured by luminex (*p<0.05, *t*-test, n=4-6 mice/group). (A-G) The graphs show data as mean±s.e.m. (Teff: effector T cell, DLN: draining lymph node)

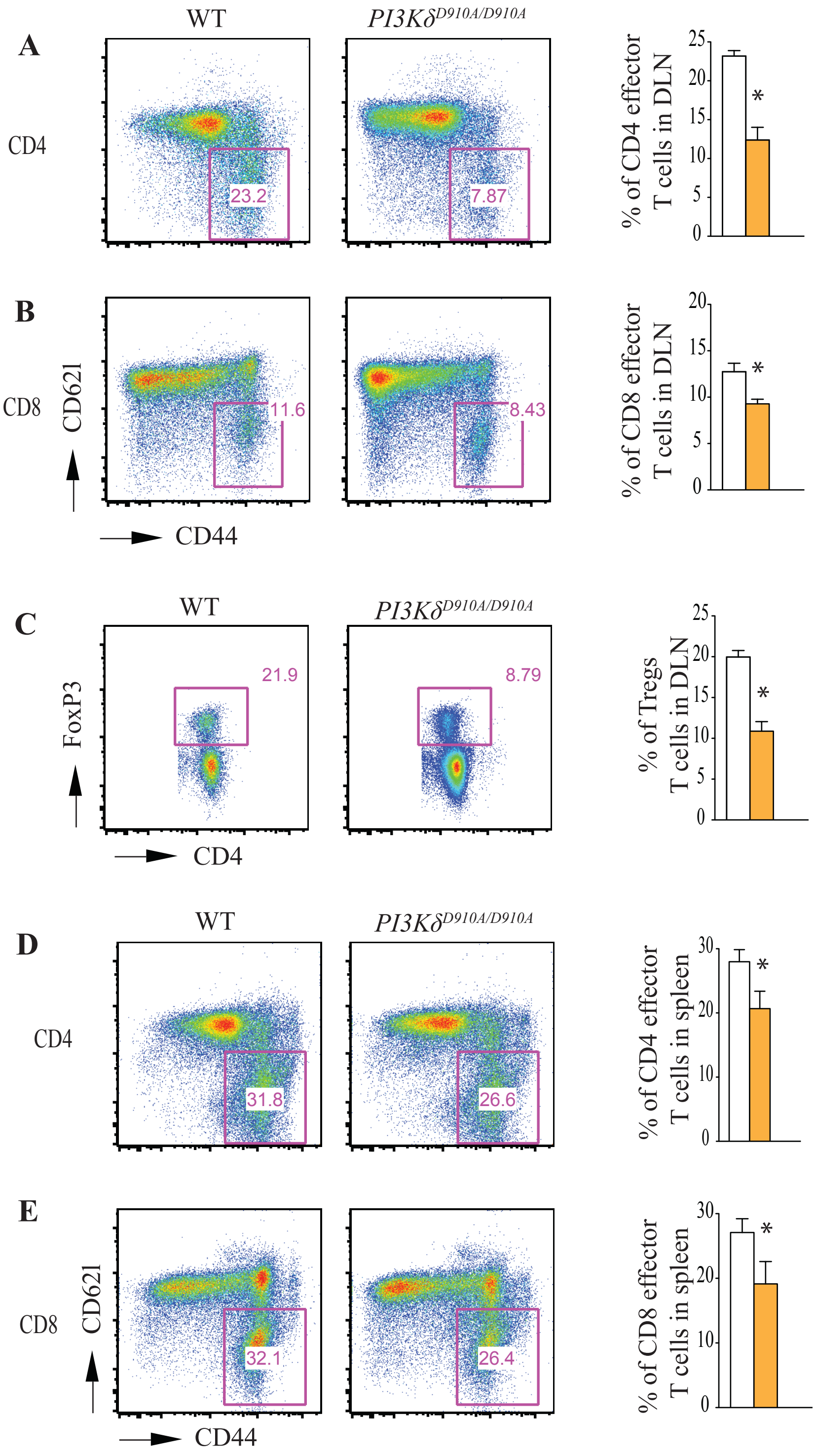

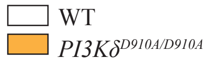

Supplementary Figure 3: PI3Kδ deletion suppresses Teff: Naïve BALB/c heart allografts were transplanted into fully allogeneic C57BL/6 (WT) or *PI3K*$\delta^{D910A/D910A}$ recipients. (A) Examples of dot plots of $CD4^+$ Teff in DLN of *PI3K*$\delta^{D910A/D910A}$ recipients compared to WT at day 7 post-transplant. Bar graph represents the percentage of $CD4^+$ Teff in DLN in *PI3K*$\delta^{D910A/D910A}$ recipients compared to WT at day 7 post-transplant (*p<0.05, *t*-test, n=6/group). (B) Examples of dot plots of $CD8^+$ Teff in DLN of *PI3K*$\delta^{D910A/D910A}$ recipients compared to WT at day 7 post-transplant. Bar graph represents the percentage of $CD8^+$ Teff in DLN in *PI3K*$\delta^{D910A/D910A}$ recipients compared to WT at day 7 post-transplant (*p<0.05, *t*-test, n=6/group). (C) Examples of dot plots of Tregs in DLN of *PI3K*$\delta^{D910A/D910A}$ recipients compared to WT at day 7 post-transplant. Bar graph represents the percentage of Tregs in DLN in *PI3K*$\delta^{D910A/D910A}$ recipients compared to WT at day 7 post-transplant (*p<0.05, *t*-test, n=6/group). (D) Examples of dot plots of $CD4^+$ Teff in splenocytes of *PI3K*$\delta^{D910A/D910A}$ recipients compared to WT at day 7 post-transplant. Bar graph represents the percentage of $CD4^+$ Teff in splenocytes in *PI3K*$\delta^{D910A/D910A}$ recipients compared to WT at day 7 post-transplant (*p<0.05, *t*-test, n=6/group). (E) Examples of dot plots of $CD8^+$ Teff in splenocytes of *PI3K*$\delta^{D910A/D910A}$ recipients compared to WT at day 7 post-transplant. Bar graph represents the percentage of $CD8^+$ Teff in splenocytes in *PI3K*$\delta^{D910A/D910A}$ recipients compared to WT at day 7 post-transplant (*p<0.05, *t*-test, n=6/group). (A-E) The graphs show data as mean±s.e.m. (Teff: effector T cell, DLN: draining lymph node, Treg: regulatory T cell)

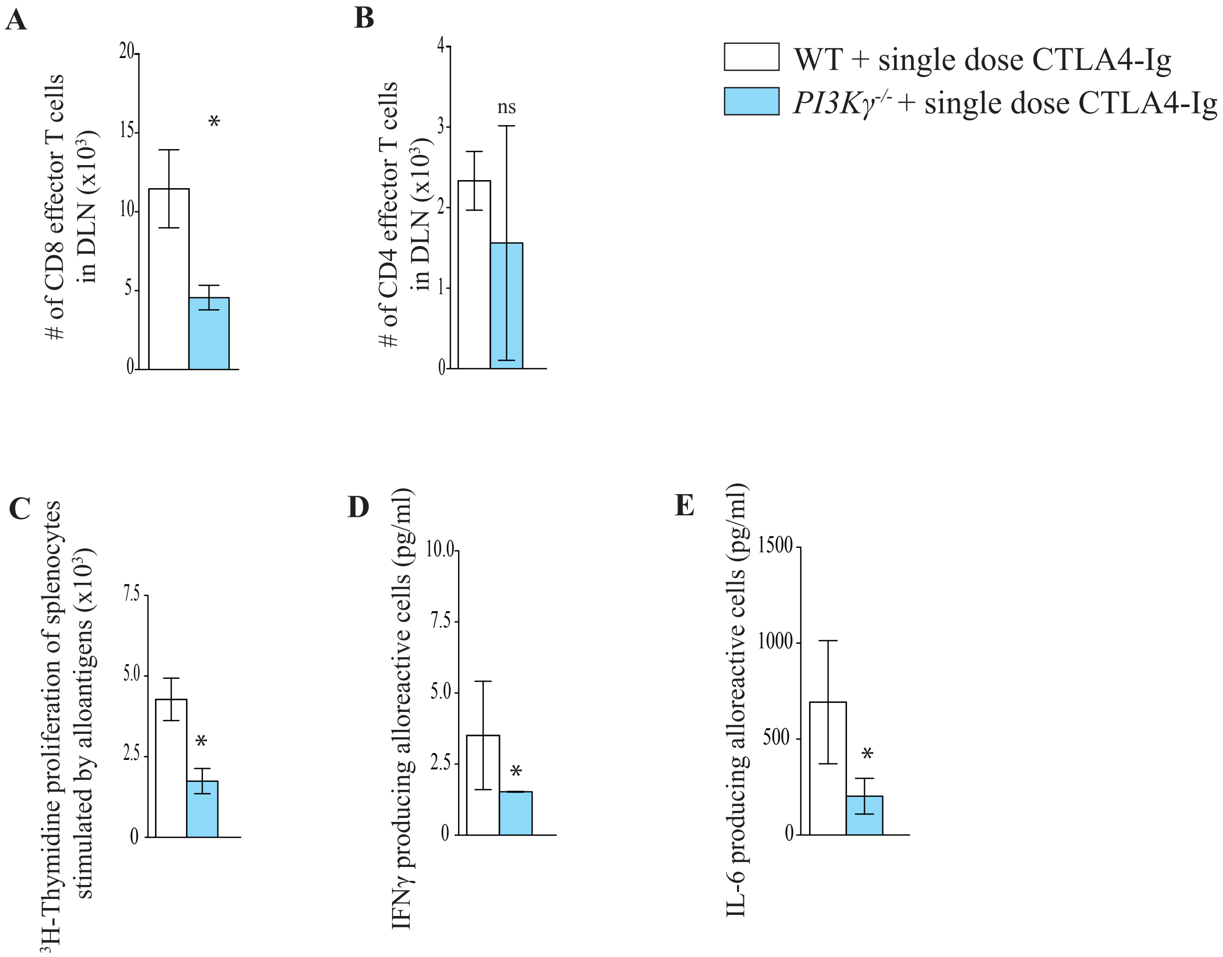

Supplementary Figure 4: Synergism of single dose CTLA4-Ig and PI3Kγ inhibition: (A) Bar graph represents the absolute count of $CD8^+$ effector T cells in the DLN of *PI3Kγ*$^{-/-}$ and WT recipients treated with single dose CTLA4-Ig (*p<0.05, *t*-test, n=6/group). (B) Bar graph represents the absolute count of $CD4^+$ effector T cells in the DLN of *PI3Kγ*$^{-/-}$ and WT recipients treated with single dose CTLA4-Ig (*p<0.05, *t*-test, n=6/group). (C) Bar graph shows that splenocytes from *PI3Kγ*$^{-/-}$ recipients treated with single dose CTLA4-Ig proliferated less upon stimulation with donor splenocytes compared to WT recipients treated with single dose CTLA4-Ig (*p<0.05, *t*-test, n=3/group). (D) Bar graph represents the level of IFNγ in the supernatant collected from the MLR assay as measured by luminex (*p<0.05, *t*-test, n=3/group). (E) Bar graph represents the level of IL-6 in the supernatant collected from the MLR assay as measured by luminex. (*p<0.05, *t*-test, n=3/group). (A-E) The graphs show data as mean±s.e.m. (DLN: draining lymph node)

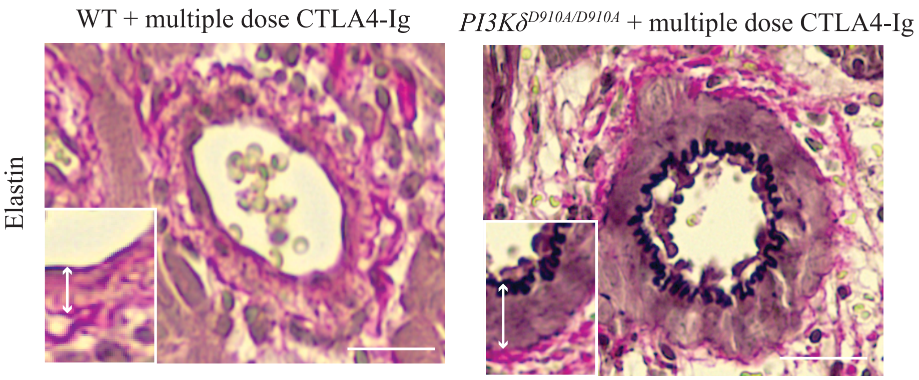

Supplementary Figure 5: Vascular intima-media thickness: Vascular intima-medial thickness was observed in cardiac allograft harvested 100 days after transplant. *PI3Kδ*$^{D910A/D910A}$ treated with multiple dose CTLA4-Ig showed thicker intima-media compared to WT treated with multiple dose CTLA4-Ig. White arrow shows intima-media thickness (Elastin stain, scale bar; 25μm).

Supplementary Figure 5

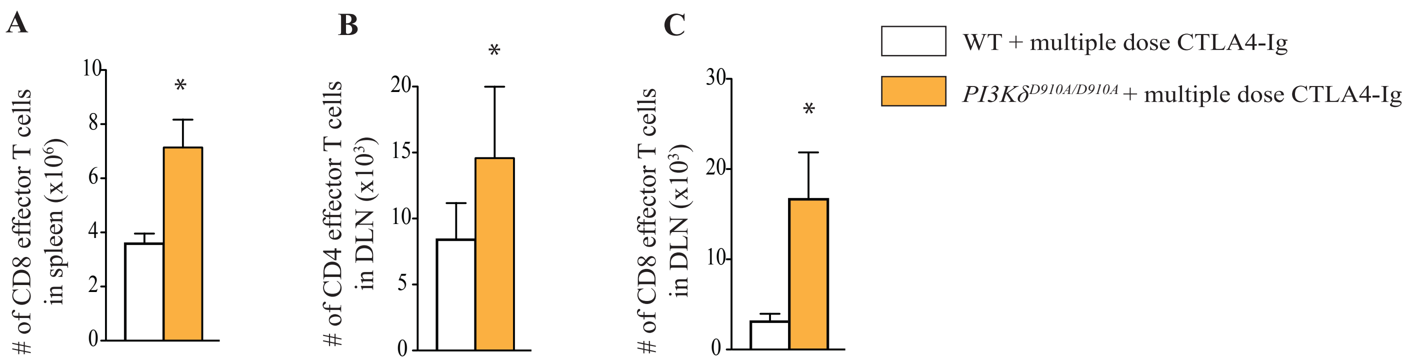

Supplementary Figure 6: PI3Kδ inhibition stimulates alloimmunity: (A) Bar graph represents the absolute number of CD8$^+$ Teff in the spleen of *PI3Kδ*$^{D910A/D910A}$ and WT recipients treated with multiple dose CTLA4-Ig at day 28 post-transplant (*p<0.05, *t*-test, n=6/group). (B) Bar graph represents the absolute number of CD4$^+$ Teff in the DLN of *PI3Kδ*$^{D910A/D910A}$ and WT recipients treated with multiple dose CTLA4-Ig at day 28 post-transplant (*p<0.05, *t*-test, n=6/group). (C) Bar graph represents the absolute number of CD8$^+$ Teff in the DLN of *PI3Kδ*$^{D910A/D910A}$ and WT recipients treated with multiple dose CTLA4-Ig at day 28 post-transplant (*p<0.05, *t*-test, n=6/group). (A-C) The graphs show data as mean±s.e.m. (Teff: effector T cell, DLN: draining lymph node)

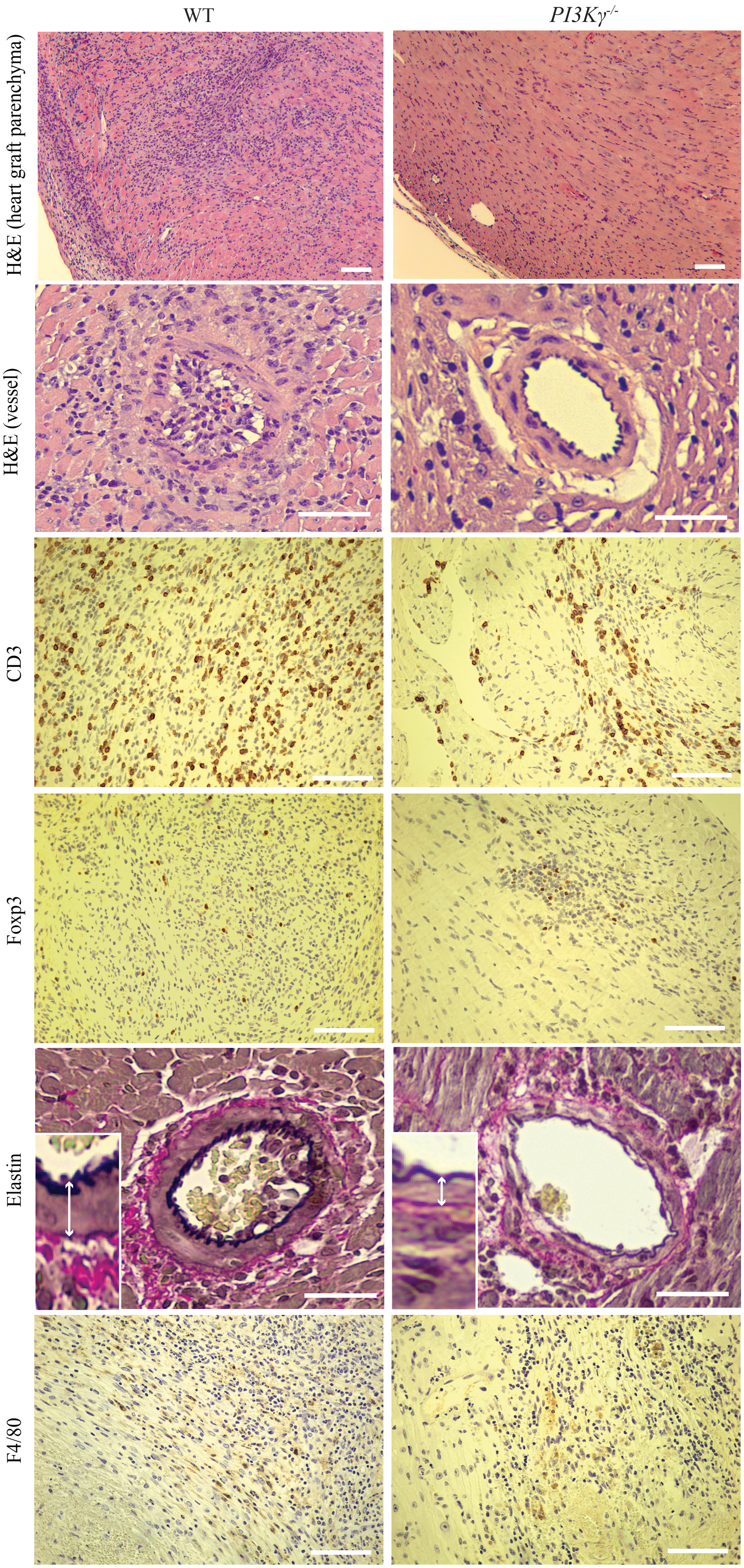

Supplementary Figure 7: PI3Kγ deletion protects heart allografts in chronic model: Heart allografts from B6.H-2[bm12] mice were transplanted into C57BL/6 (WT) or *PI3Kγ*$^{-/-}$ mice and removed from recipients at day 28 post-transplant for histological assessment. Heart allograft histology showed less cellular, CD3 and F4/80 infiltration, and intima-medial thickness in the *PI3Kγ*$^{-/-}$ recipients group compared to WT controls (Scale bar, 50μm for H&E heart graft parenchyma, CD3, Foxp3 and F4/80. Scale bar; 25μm for H&E vessel and Elastin stain. White arrow in elastin staining shows intima-medial thickness).

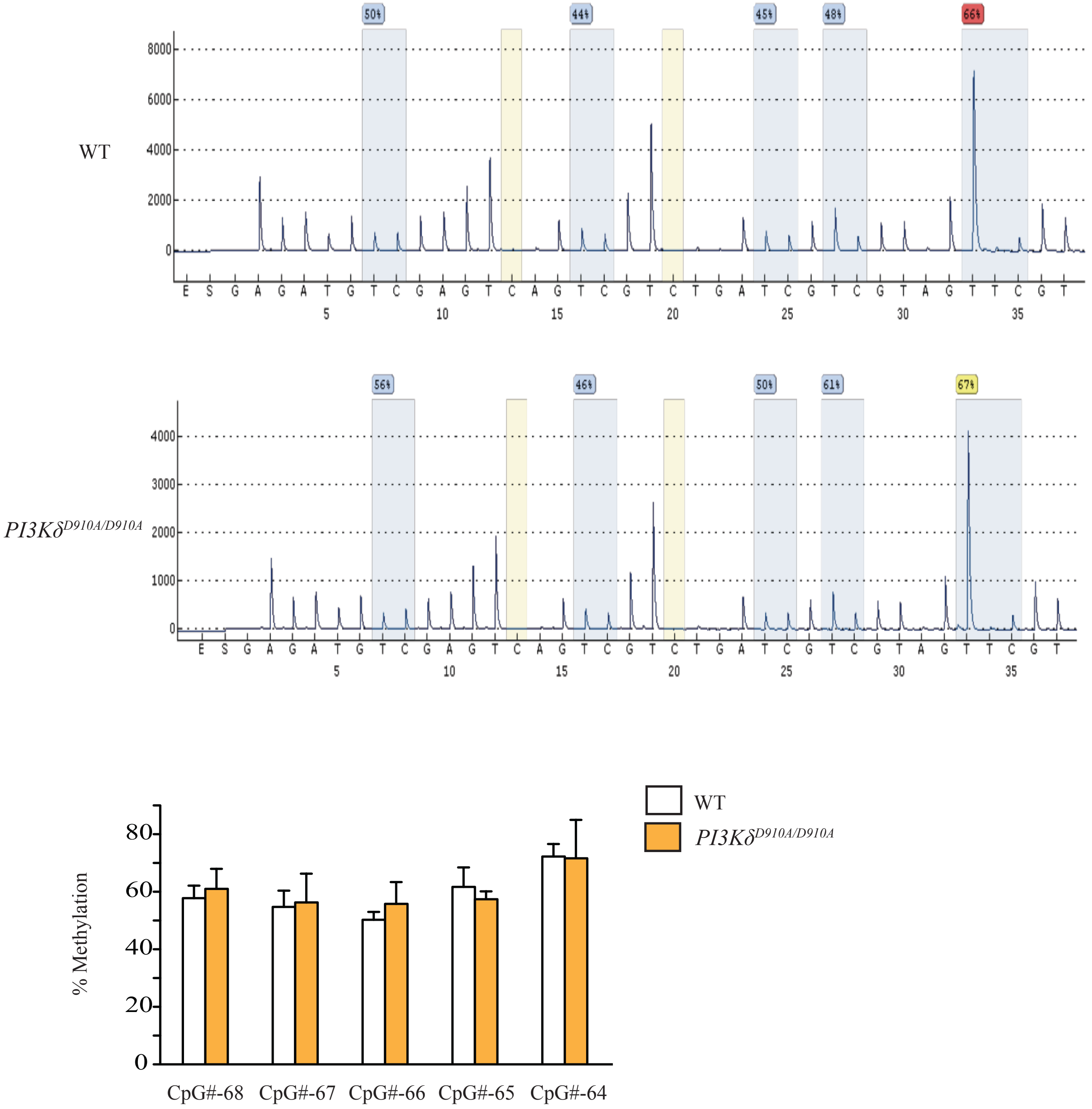

Supplementary Figure 8: PI3Kδ deletion has no effect on *FoxP3* methylation: Representative examples of the gene methylation analysis at the five CpG sites of the proximal promoter of *FoxP3* (location: -6750 to -6714 from *ATG*) of Tregs isolated from WT and $PI3K\delta^{D910A/D910A}$ mice and stimulated *in vitro* for 24 hours. Bar graph quantifies the methylation at each of the five CpG sites (*p*=not significant, *t*-test, n=3/group, the graphs show data as mean±s.e.m.). (Treg: regulatory T cell)

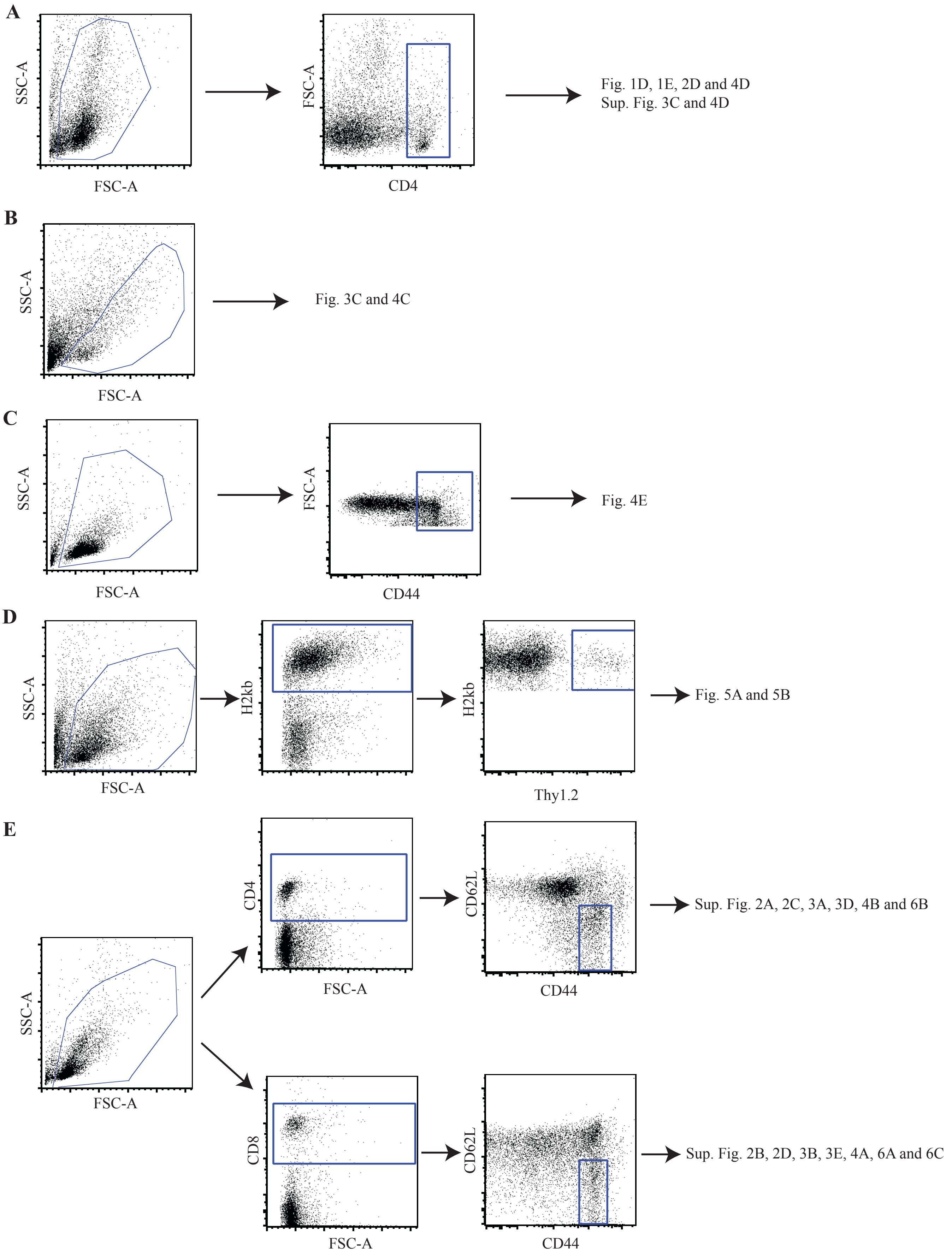

Supplementary Figure 9: FACS gating strategies: (A) Sequential gating strategy for $CD4^+$ $Foxp3^+$ T cells related to Figure 1D, 1E, 2D, 4D, Supplementary Figure 3C and 4D. (B) Sequential gating strategy for $CD4^+$ or $CD8^+$ T cells related to Figure 3C and 4C. (C) Sequential gating strategy for $CD8^+$ $CD44^{high}$ GrB^+ T cells related to Figure 4E. (D) Sequential gating strategy for Annexin or Ki67 $CD4^+$ $Foxp3^+$ T cells related to Figure 5A and 5B. (E) Sequential gating strategy for $CD4^+$ or $CD8^+$ T effector memory cells ($CD62L^-$ $CD44^{high}$) related to Supplementary Figure 2A-D, 3A-B, 3D-E, 4A-B and 6A-C.
